# The Impact of Divergence Time on the Nature of Population Structure: An Example from Iceland

**DOI:** 10.1371/journal.pgen.1000505

**Published:** 2009-06-05

**Authors:** Alkes L. Price, Agnar Helgason, Snaebjorn Palsson, Hreinn Stefansson, David St. Clair, Ole A. Andreassen, David Reich, Augustine Kong, Kari Stefansson

**Affiliations:** 1 Department of Epidemiology, Harvard School of Public Health, Boston, Massachusetts, United States of America; 2 Department of Biostatistics, Harvard School of Public Health, Boston, Massachusetts, United States of America; 3 Broad Institute of MIT and Harvard, Cambridge, Massachusetts, United States of America; 4 deCODE Genetics, Reykjavik, Iceland; 5 University of Iceland, Reykjavik, Iceland; 6 Department of Mental Health, University of Aberdeen, Aberdeen, United Kingdom; 7 Division of Psychiatry, Ulleval University Hospital, Oslo, Norway; 8 Department of Genetics, Harvard Medical School, Boston, Massachusetts, United States of America; University of Oxford, United Kingdom

## Abstract

The Icelandic population has been sampled in many disease association studies, providing a strong motivation to understand the structure of this population and its ramifications for disease gene mapping. Previous work using 40 microsatellites showed that the Icelandic population is relatively homogeneous, but exhibits subtle population structure that can bias disease association statistics. Here, we show that regional geographic ancestries of individuals from Iceland can be distinguished using 292,289 autosomal single-nucleotide polymorphisms (SNPs). We further show that subpopulation differences are due to genetic drift since the settlement of Iceland 1100 years ago, and not to varying contributions from different ancestral populations. A consequence of the recent origin of Icelandic population structure is that allele frequency differences follow a null distribution devoid of outliers, so that the risk of false positive associations due to stratification is minimal. Our results highlight an important distinction between population differences attributable to recent drift and those arising from more ancient divergence, which has implications both for association studies and for efforts to detect natural selection using population differentiation.

## Author Summary

The Icelandic population is a structured population, in that geographic regions of Iceland exhibit differences in allele frequencies of genetic markers. Although these differences are relatively small, previous work has shown that they can bias association statistics in disease studies if cases and controls are sampled in different proportions across the geographic regions. In this study, we show that by using dense genotype data it is possible to distinguish the regional geographic ancestry of individuals from Iceland. We further show that the allele frequency differences between regions of Iceland are due to genetic drift since the settling of Iceland, not to differences in contributions from ancestral populations. A consequence of this is that the allele frequency differences follow a null distribution, devoid of unusually large differences caused by the action of natural selection, so that ensuing false positive associations in disease studies will be minimal. This is in stark contrast to populations (such as European Americans) in which subpopulation differences are due to more ancient divergence, allowing the action of natural selection to produce unusually large allele frequency differences that can lead to false positive associations. Our results highlight an important distinction between population differences attributable to recent genetic drift and those arising from more ancient divergence.

## Introduction

The Icelandic population has been sampled in many disease association studies [1]–[8]. Thus, there is a strong motivation to understand the structure of this population and the ramifications for association studies. A recent study of 40 microsatellite markers showed that the Icelandic population is relatively homogeneous, but that subtle subpopulation differences exist, inflating disease association statistics in simulated case-control studies [9]. Other studies of Icelandic population structure have focused on Y chromosome and mtDNA analyses [10]–[12]. Now, the availability of genotype data from a large number of Icelandic samples, based on densely distributed SNPs from all over the genome and collected in the course of genome-wide association studies, makes it possible to investigate Icelandic population structure in greater depth. In this study, we analyzed over 30,000 Icelandic samples that were genotyped using the Illumina 300 K chip.

In addition to providing a more detailed assessment of genetic differences between regional subpopulations, our analyses yield several new results. First, we show that with a sufficient amount of genotype data it is possible to distinguish regional geographic ancestries of individuals from Iceland, and to demonstrate a striking concordance between genetic relationships and Icelandic geography. Second, we show that population structure in Iceland is due to recent genetic drift, not to regional differences in the proportion of admixture from Norse and Gaelic ancestral populations [11]. Third, we show that allele frequency differences between regional subpopulations follow a null distribution that is devoid of highly differentiated SNPs, consistent with the young age of the Icelandic population. A noteworthy consequence is that there is minimal risk of confounding due to population stratification in association studies performed in Iceland. This is in stark contrast to differences among populations of European ancestry (e.g., as represented in European Americans [13],[14]), where, even in the face of low levels of aggregate population differentiation, confounding can arise from unusually differentiated loci that are the result of geographically restricted episodes of natural selection during much longer periods of population divergence. Indeed, a genetic comparison of Icelanders and Scots revealed an excess of highly differentiated variants, including variants for which the unusual extent of differentiation was genomewide-significant, suggesting the action of natural selection. Thus, both the curse of population stratification and the blessing of using unusually differentiated loci to detect natural selection are far more pertinent in populations with a subtle level of structure arising from ancient divergence than in populations such as that of Iceland whose subtle structure is the result of recent genetic drift.

## Results

### Genetic Relationships between 11 Regions of Iceland

**Figure 1:**
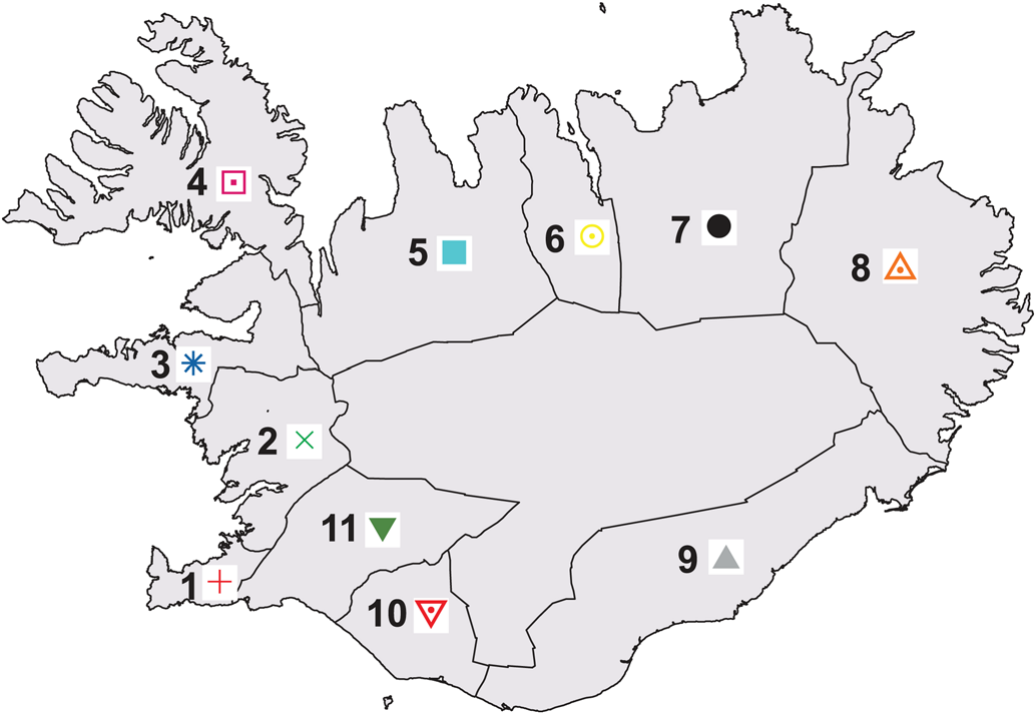
Map of 11 regions of Iceland, color-coded to match Figures 2 and 3. The interior region is not numbered, as it is uninhabited. Sample sizes for each region are listed in Table 1.

During the past century, urbanization has led to considerable mixing of ancestry from the different regions of Iceland, particularly in the capital city of Reykjavik [9]. However, our aim here was to study the population structure as it existed prior to this mixing. To this end, our initial analyses focused on a subset of 877 Icelandic samples of over 30,000 that were genotyped on the Illumina 300 K chip. For each of 11 regions of Iceland, we chose up to 100 unrelated samples with majority ancestry from that region, based on genealogical information from their ancestors five generations back (Figure 1 and Table 1; see Materials and Methods).

**Table 1:** Data for Icelandic samples with majority ancestry from each of the 11 regions.

Region	1	2	3	4	5	6	7	8	9	10	11
Total	47	959	1154	3667	1343	1108	1102	1368	803	1447	1315
Unrelated	3	55	65	100	100	100	98	100	61	100	95

For each region, we list the total number of Icelandic samples with majority ancestry from that region, and the number of unrelated samples that were selected.

**Figure 2:**
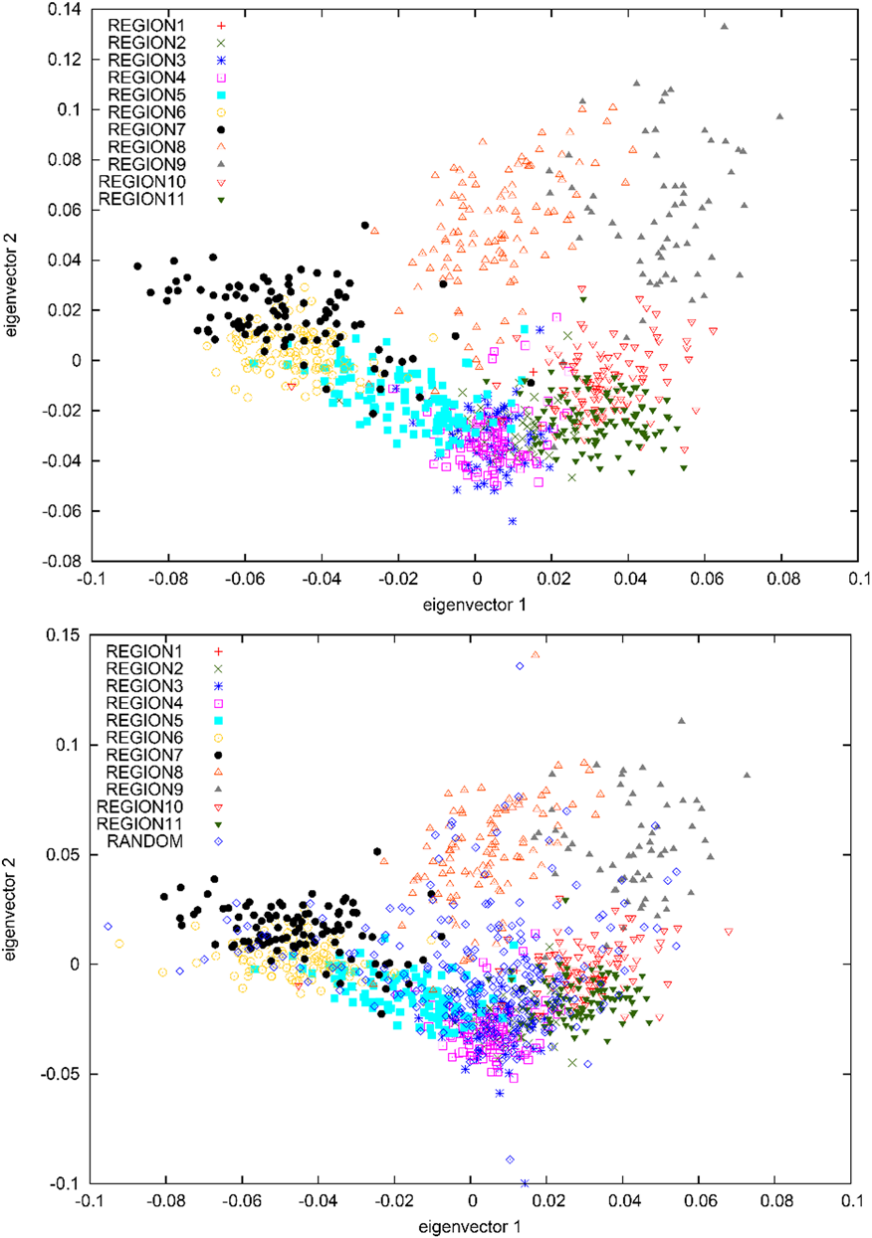
PCA plots of (A) samples with most of their ancestry from 11 regions of Iceland and (B) samples with most of their ancestry from 11 regions of Iceland, together with a set of 250 randomly selected Icelandic samples.

Principal components analysis (PCA) is a widely used tool for analyzing genetic data [15]–[18]. We ran PCA on genotype data from the 877 individuals using the EIGENSOFT software with default parameters settings [17]. A plot of the top two principal components is displayed in Figure 2A, revealing a striking concordance between the geographical orientation of the 11 regions (Figure 1) and the relative positions of each region on the PCA plot (Figure 2A). In both cases, we observe a ring-shaped topology with region numbers increasing in clockwise order and a central void corresponding to the unpopulated interior of Iceland. The top two PCs explain a modest proportion of the overall variance: 0.0027 for PC1 and 0.0022 for PC2, representing an excess of 0.0015 for PC1 and 0.0011 for PC2 above what would be expected by chance (Tracy-Widom P-values<10^−12^ in each case [17]), similar to previous results on European American data sets [13]. We note that these PCs are the result of genome-wide structure, as opposed to a small number of highly informative markers (see Text S1).

To evaluate the use of dense genotype data to predict geographic ancestry in the Icelandic population, we randomly selected 250 additional Icelandic samples for which genotype data was available (see Materials and Methods). A PCA run with the 250 samples included (Figure 2B) indicates that these individuals trace their ancestry from all over Iceland, with an excess of individuals from the vicinity of region 4 (concordant with Table 1). We used the PCA results to predict the regional ancestry of each of the 250 samples and compared this with their true ancestry, which we defined as the region in which the greatest number of ancestors five generations back was born (see Materials and Methods). The ancestry predictions were correct for 47% of samples, correct to within a distance of one region for 74% of samples, and correct to within a distance of two regions for 93% of samples. The accuracy increased to 58% (87% to within one region, 97% to within two regions) when restricting to the 98 (of 250) samples with at least 16 of 32 ancestors from a single region. Our analyses demonstrate that dense genotype data can be used to distinguish, and to some extent predict, the regional geographic ancestry of individuals within Iceland. We note that a correlation between geography and genetic ancestry has also been observed in other parts of Europe [19]–[22].

A different way to examine the patterns of genetic variation in Iceland is through summary statistics such as *F_ST_*, which reflects the proportion of the total genetic variation found in two populations that is explained by their division into separate populations [23],[16] (see Materials and Methods). *F*_ST_ values were computed for each pair of Icelandic regions, yielding an average of 0.0026 (Table 2). Both Figure 2A and Table 2 show that region 7 and particularly region 9 show the greatest divergence from the other regions, as well as the lowest heterozygosity, which suggests that these regions have been more influenced by genetic drift than the others. This finding is consistent with the small historical population sizes of these regions [24].

**Table 2:** Pairwise *F*_ST_ and heterozygosity estimates for 11 regions of Iceland.

*F*_ST_	1	2	3	4	5	6	7	8	9	10	11
1	0.3505	0.0019	0.0024	0.0022	0.0021	0.0031	0.0036	0.0032	0.0042	0.0018	0.0022
2		0.3479	0.0013	0.0015	0.0016	0.0027	0.0030	0.0027	0.0038	0.0018	0.0019
3			0.3475	0.0012	0.0015	0.0027	0.0030	0.0027	0.0040	0.0021	0.0022
4				0.3478	0.0014	0.0027	0.0028	0.0027	0.0039	0.0020	0.0023
5					0.3474	0.0014	0.0021	0.0024	0.0039	0.0020	0.0023
6						0.3468	0.0018	0.0030	0.0048	0.0031	0.0034
7							0.3457	0.0029	0.0049	0.0033	0.0035
8								0.3466	0.0032	0.0025	0.0030
9									0.3446	0.0027	0.0036
10										0.3479	0.0012
11											0.3470

Heterozygosity values are listed on the diagonal. Standard errors of *F*_ST_ estimates were equal to 0.0007 for all comparisons involving Region 1 and 0.0001 for all other comparisons.

### Genetic Relationships between Iceland, Norway, and Scotland

The Icelandic population arose from the admixture of Norse and Gaelic ancestors around 1100 years ago, at the time of settlement [11]. Pairwise *F*_ST_ values between Iceland, Norway and Scotland were computed based on the 79,641 autosomal SNPs in the intersection of the Illumina 300 K and Affymetrix 6.0 chips, using genotype data from 30,244 Icelandic, 250 Norwegian and 445 Scottish samples (see Materials and Methods). The resulting *F*_ST_ estimates were 0.0016 between Iceland and Norway, 0.0020 between Iceland and Scotland, and 0.0013 between Norway and Scotland. The larger *F*_ST_ estimates separating Iceland and its two ancestral populations are consistent with previous analyses indicating that the Icelandic gene pool has experienced more recent drift than neighboring countries in northern Europe [12].

**Figure 3:**
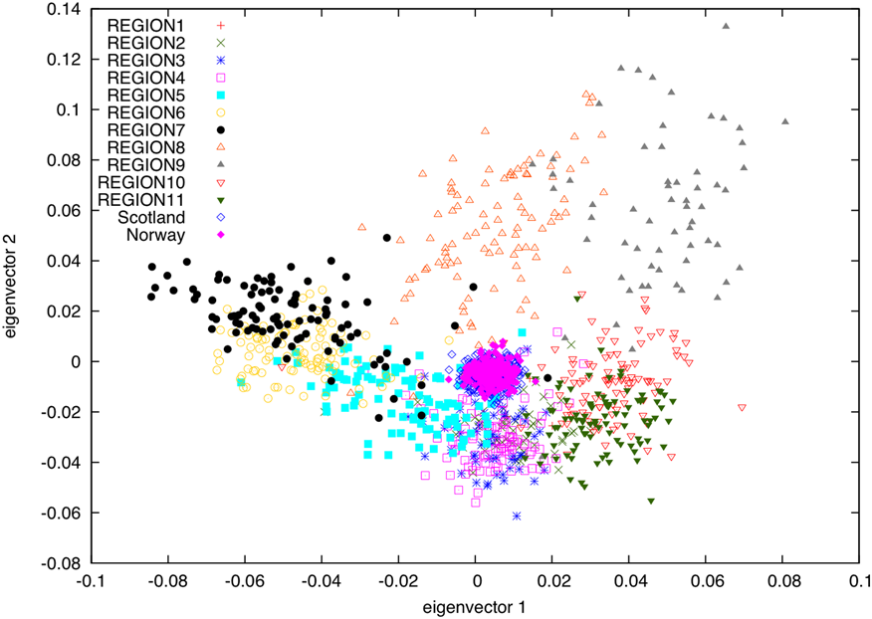
PCA plot of samples from Norway and Scotland projected onto PCs computed using samples with most of their ancestry from 11 regions of Iceland.

One possible explanation for the genetic differences observed between the 11 regions of Iceland is varying contributions from ancestral populations. To explore this possibility, we used genotypes from the 79,641 overlapping SNPs to project [17] the Norwegian and Scottish samples onto principal components computed using the subset of 877 Icelandic samples (Figure 3). This analysis is robust to the concern that projected samples may be affected by regression towards the mean (see Text S1, Figure S1, and Figure S2). The Norwegian and Scottish samples were tightly clustered near the origin, with each having a mean of 0.004 on PC1 and −0.005 on PC2. This indicates that the genetic differences between Icelandic subpopulations represented on the top two PCs are orthogonal to genetic differences between the Norwegian and Scottish ancestral populations. In other words, varying contributions from ancestral populations are not a major determinant of genetic differences between Icelandic regions. Rather, the most plausible source of these differences is genetic drift during the 1100 years that have passed since the settlement of Iceland.

### Estimating the Norse and Gaelic Contributions to Icelandic Ancestry

To obtain a direct estimate of Norse and Gaelic ancestry proportions in the Icelandic population, we modeled Icelandic allele frequencies as a linear combination of Norwegian and Scottish allele frequencies, accounting for the sampling error arising from the limited sample sizes (see Materials and Methods). While the Norwegian and Scottish samples may not perfectly represent the ancestral populations of Icelandic settlers—who derived from several parts of Norway, possibly other parts of Scandinavia, Scottish coastal regions and Ireland—we postulated that they were close enough to provide a reasonable admixture estimate. Based on the available data, the optimal linear combination yielded an estimate of 64% Norse and 36% Scottish ancestry, with a standard error of less than 2%. The *F*_ST_ between the optimal linear combination and the observed allele frequencies in Iceland was 0.0014, which may be in part due to inadequate sampling from the true ancestral populations, but is likely to be mainly due to recent genetic drift in the Icelandic gene pool.

The same computation was performed for each of the 11 Icelandic regions, yielding ancestry estimates that were not statistically different. For each region, the estimate of Norse ancestry was between 62% and 65%, with a standard error of less than 2% (except region 1, for which we obtained 61% with a standard error of less than 3%). This provides strong evidence that the proportions of Norse and Gaelic ancestry do not vary among Icelandic regions, supporting the notion that differences between Icelandic regions are due to recent genetic drift rather than varying contributions from ancestral populations.

A separate question is whether the proportion of Norse ancestry was greater among male settlers of Iceland than among female settlers, as previous studies based on Y-chromosome and mtDNA haplotypes have suggested [10],[11]. A comparison of ancestry estimates for X-chromosome vs. autosomal SNPs could potentially provide an answer to this question, since two-thirds of X-chromosome alleles (vs. one-half of autosomal alleles) are passed through the female line. We obtained an X-chromosome ancestry estimate of 63% Norse and 37% Scottish ancestry, with a standard error of 7%. The standard error was quite large—our analysis was limited to only 2,962 X-chromosome SNPs present on both the Illumina 300 K and Affymetrix 6.0 chips—and hence this analysis is inconclusive. Because ancestry differences between the X chromosome and autosomes would be expected to be much smaller than the underlying ancestry effects (for example, a 100% difference between the ancestry of male settlers and female settlers would lead to an X-chromosome vs. autosome ancestry difference of only 17%), our results do not contradict the hypothesis of a substantial ancestry difference between male and female settlers.

### Distribution of Allele Frequency Differences between Icelandic Subpopulations

**Figure 4:**
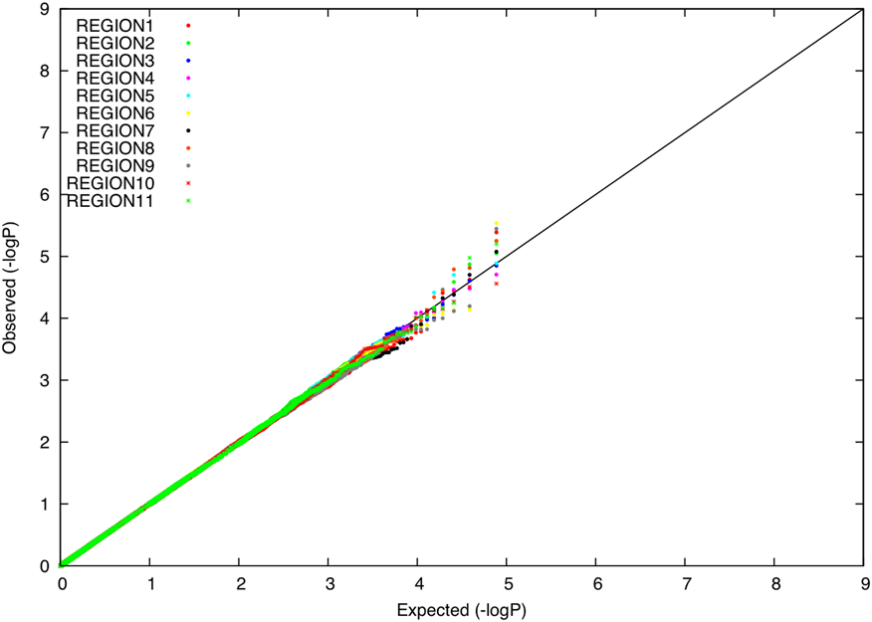
P-P plots of allele frequency differentiation between region *r* and the union of all other regions, for each value of *r* (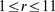).

We evaluated whether there is an excess of common SNPs with large allele frequency differences between Icelandic subpopulations, using data from 14,313 individuals with majority ancestry from one of 11 Icelandic regions (Table 1). For each Icelandic region, we computed the distribution of allele frequency differences between that region and the union of all other regions, expressed as a χ^2^ (1 d.o.f.) statistic under a model of neutral genetic drift. This computation accounts for related individuals (see Materials and Methods). P-P plots for each region *r* (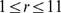) are displayed in Figure 4. For each region, there was no excess of markers with large frequency differences versus other regions. Averaging across computations for each of 11 regions, 0.008% of markers had a P-value less than 0.0001, roughly matching the expected distribution. The most significant P-value was 3×10^−6^, a value that is not statistically significant after correcting for the number of SNPs and regions tested. These results are consistent with the hypothesis that the divergence time of Icelandic regions has been too short for differential selective forces to have had a significant impact on allele frequencies.

In a disease association study where cases and controls are drawn from distinct populations, there is a mathematical relationship between the distribution of allele frequency differences and the distribution of disease association statistics (see Materials and Methods). We obtained empirical agreement with this theoretical result by simulating a case-control study in which 100 unrelated samples with majority ancestry from region 4 were labeled as disease cases and 100 unrelated samples with majority ancestry from region 5 were labeled as controls. We computed Cochran-Armitage trend statistics and obtained a genomic control *λ* of 1.285, consistent with the predicted value of (1+*NF*_ST_) = 1.28 given the *F*_ST_ of 0.0014 between the two regions (see Materials and Methods). After dividing by Cochran-Armitage trend statistics by the genomic control *λ*, the most significant association had a P-value of 3×10^−6^, which is not statistically significant after correcting for the number of SNPs tested. We repeated this analysis for all pairs of regions (4,5,6,8,10) with 100 unrelated samples available (see Table 1), and obtained similar results (minimum P-value of 4×10^−7^, which is not statistically significant after correcting for the number of SNPs and number of pairs of regions tested.)

A consequence of these findings is that whenever *λ* is close to 1 in a disease association study involving the Icelandic population, false positive associations due to population stratification can be conclusively ruled out. If *λ* is greater than 1, then dividing association statistics by *λ* will still prevent false positive associations. This is not the case in populations, such as European Americans, with a subtle level of structure arising from more ancient divergence [25].

### Distribution of Allele Frequency Differences between Iceland and Scotland

**Figure 5:**
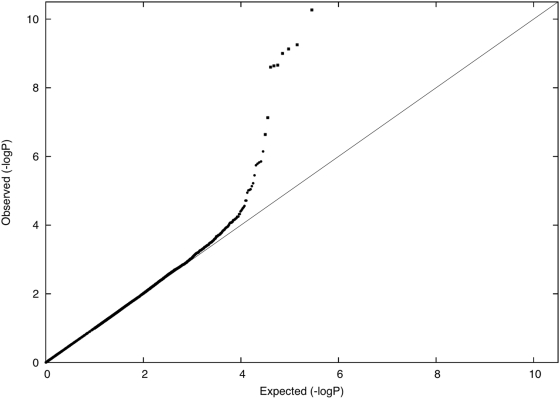
P-P plot of allele frequency differentiation between Norway and Scotland. The nine SNPs from Table 3 are displayed as squares.

We evaluated whether an excess of common SNPs with large allele frequency differences between Icelanders and Scots could provide evidence of population-specific natural selection. We used Icelanders and Scots (rather than Norwegians) in this analysis, because these samples were genotyped on the same chip under identical assay conditions, thus avoiding the effects of differential bias [26]. Indeed, tail distributions of comparisons between populations genotyped on different chips appear to be confounded by assay artifacts, precluding robust analyses of those comparisons (see Text S1). We used allele frequency differences between the Icelandic and Scottish samples at common SNPs to compute a χ^2^ (1 d.o.f.) statistic for unusual population differentiation that accounts for the effects of neutral genetic drift (see Materials and Methods). A P-P plot of our results is displayed in Figure 5. In contrast to Figure 4, there is a substantial excess of markers in the extreme tail, with 0.018% of markers having a P-value less than 0.0001. We speculate that many of these markers are likely to have been under natural selection.

We found eight SNPs, representing two chromosomal regions, for which the evidence of unusual population differentiation was genomewide-significant (nominal P-value<10^−7^, P-value<0.03 after correcting for 284,191 common SNPs tested). Six of the SNPs lie in or near the *TLR* (toll-like receptor) genes *TLR*10 and *TLR*1, while the other two lie inside the *NADSYN*1 (NAD synthesase 1) gene (http://genome.ucsc.edu/) (Table 3). For each of these SNPs, the allele frequency difference between Icelanders and Scots was greater than 15% (Table S1), far in excess of typical allele frequency differences of about 3% that correspond to an *F*_ST_ value of 0.0020. Only two of the SNPs from Table 3 were present in Norwegian data based on the Affymetrix 6.0 chip (rs10024216 and rs11096957 in the *TLR* region), but for both of these SNPs—and also for rs7940244 in the *NADSYN1* region (which was not genomewide-significant in the comparison of Icelanders and Scots)—allele frequency differences between Norwegians and Scots were likewise greater than 15% (Table S1), ruling out an effect specific to Icelanders. We also report frequencies of these SNPs in HapMap populations [27] (Table S1). We note that both *TLR* and *NADSYN*1 were previously reported to be significantly differentiated among 12 British subpopulations analyzed by the WTCCC (nominal P-values of 10^−12^ for *TLR* and 10^−8^ for *NADSYN1*) [28]. The WTCCC study has made an important and valuable contribution to research on natural selection by highlighting the potential utility of large sample sizes from very closely related populations for detecting signals of selection. However, the statistical test employed by those authors only evaluated whether frequency differences between the 12 subpopulations were different from zero, and not whether the amount of differentiation was in excess of what would be expected under neutral genetic drift (as inferred from genome-wide patterns). As an illustration of this distinction, we observed that a total of 3,982 SNPs in our data set had frequency differences between Iceland and Scotland that were different from zero at the nominal P-value threshold of 10^−7^ used for the corresponding test in the WTCCC study. It is extremely unlikely that all of these SNPs were under selection. Thus, it is not possible to conclude whether the results of the WTCCC study represent genomewide-significant signals of selection. However, our findings support the hypothesis that selection did occur.

**Table 3:** List of markers whose unusual differentiation between Iceland and Scotland is genomewide-significant.

Marker	Chromosome	Build35 Position	Nominal P-Value	Inside Gene?
rs10024216	4	38,586,678	7×10^−8^	
rs10008492	4	38,588,286	7×10^−10^	
rs4331786	4	38,591,974	2×10^−9^	
rs11096957	4	38,599,057	1×10^−9^	*TLR10*: exon
rs4543123	4	38,615,090	5×10^−11^	
rs4833095	4	38,622,276	6×10^−10^	*TLR1*: exon
rs7944926	11	70,843,273	2×10^−9^	*NADSYN1*: intron
rs3794060	11	70,865,327	3×10^−9^	*NADSYN1*: intron
rs13107325*	4	103,545,887	2×10^−7^	*SLC39A8*: exon

A total of 12 markers in the *TLR* region and 5 markers in the *NADSYN1* region achieved a nominal P-value of 0.0001 or lower (data not shown). We list with an asterisk one additional marker whose differentiation is highly suggestive (see text). Gene names are listed for markers located between the transcription start and end sites of a gene.

In addition to the eight genomewide-significant signals, a highly suggestive signal of unusual differentiation was observed at the SNP rs13107325 (nominal P-value = 2×10^−7^, P-value = 0.06 after correcting for 284,191 common SNPs tested) (Table 3). This SNP is a missense coding SNP inside the *SLC39A8* (solute carrier family 39 (zinc transporter), member 8) gene (http://genome.ucsc.edu/), and allele frequencies in HapMap [27] indicate that the minor allele of this SNP is private to populations of European ancestry (Table S1). Thus, although this SNP did not meet our strict criteria for genome-wide significance, it is an intriguing candidate for natural selection.

## Discussion

We analyzed the population structure of Iceland using dense genotype data to show that there are subtle but discernable genetic differences between individuals from different Icelandic regions, and that these differences are broadly consistent with the ring-shaped topology of the inhabited part of Iceland. The average pairwise *F*_ST_ of 0.0026 for the 11 regions we analyzed is similar to *F*_ST_ values between different European populations. However, it is important to point out that *F*_ST_ values in this study may be heavily dependent on the sampling scheme, and *F*_ST_ values of a similar magnitude might be observed within other European countries if analyzed at the same geographical resolution. Notably, Icelandic subpopulation differences are due to recent genetic drift and not to varying contributions from ancestral populations, as the subpopulations from each Icelandic region inherit roughly 64% Nordic and 36% Gaelic ancestry.

A consequence of the recent origin of the genetic differences between Icelandic subpopulations is that allele frequency differences follow the null distribution predicted by neutral drift. Thus, there is little risk of false positive associations due to population stratification in disease association studies, despite the fact that there are genuine differences between regions. The same conclusion may be expected for other populations whose structure has arisen from recent genetic drift [29]. On the other hand, such populations are not well-suited for the detection of regionally specific natural selection reflected in unusual differences between subpopulations. For that purpose, subtly structured populations whose structure is due to more ancient population divergence, with large population sizes minimizing subsequent genetic drift, offer the greatest promise. For example, European American subpopulations exhibit unusual differences at the *LCT*, *HLA* and *OCA2* loci that lie outside the null distribution with genome-wide significance ([13] and A.L. Price, unpublished data). The distinction between population differences attributable to recent drift and those arising from more ancient divergence is also likely to be of interest in studies of other subtly structured populations [22],[28],[30].

For some diseases in Iceland, such as breast cancer, the geographical distribution of patients and their ancestors is not random [31]. Our results indicate that highly differentiated common variants are unlikely to be the cause of this phenomenon. Rare variants that have risen to higher frequency in certain regions of Iceland due to founder effects provide a more plausible explanation. An example in the case of breast cancer is the *BARD1* Cys557Ser risk variant that rose in frequency in the easternmost county of Sudur-Mulasysla (Figure 1) due to a population bottleneck in that region [32]. A direction of research that is motivated by our findings is to investigate the extent to which rare variants, spread by recent founder effects, play a role in differences in disease prevalence among individuals with ancestry from different regions of Iceland.

## Materials and Methods

### Ethics Statement

This research was approved by the Data Protection Commission of Iceland and the National Bioethics Committee of Iceland. The appropriate informed consent was obtained for all sample donors.

### Icelandic Data

DNA samples from 35,457 individuals residing in Iceland were genotyped using the Illumina 300 K chip in the course of disease association studies conducted by deCODE Genetics. The appropriate informed consent was obtained for all sample donors. Owing to the sensitive nature of genotype data, access to this data can only be granted at the headquarters of deCODE Genetics in Iceland. SNPs with >5% missing data were removed, leaving 292,289 autosomal SNPs for analysis. No linkage disequilibrium or low frequency SNP filters were applied. For each Icelandic sample genotyped, additional data were available from a genealogical database describing relatedness to other samples and listing the birth county in Iceland of each ancestor tracing back five generations [33]. This information was used to restrict some analyses to subsets of Icelandic samples (see below).

### Samples with Ancestry from 11 Regions of Iceland

We grouped the 21 counties of Iceland into 11 regions, as previously described [9] (Figure 1). From the entire set of 35,457 individuals, we selected a subset of 14,313 individuals with majority ancestry from one of the 11 regions, based on having at least 16 of 32 ancestors (five generations back) from that region (Table 1a). The goal of this scheme was to choose a set of samples reflecting the population structure of Iceland prior to the large-scale migration that resulted from industrialization and urbanization during the past century. From this set of 14,313 individuals we selected a further subset of 885 individuals—with at most 100 individuals from each region—that were unrelated at a meiotic distance of four generations. Of the 885 individuals, 8 were removed as genetic outliers when we ran PCA [17]; Table 1b and subsequent analyses are based on the remaining 877 individuals. The size limit of 100 individuals was used to ensure a relatively even representation of regions for analyses that are sensitive to varying sample sizes from subpopulations. We note that region 1, which contains the capital city of Reykjavik, was heavily underrepresented as it had a small population prior to urbanization.

### An Additional 250 Icelandic Samples

We randomly selected 250 samples from the 35,457 samples that were genotyped on the Illumina 300 K chip. Of these 250 samples, five overlapped the previous set of 877 samples; these were retained in the set of 250 additional samples but excluded from the set of original samples, in which only 872 samples were retained. We ran PCA on the combined set of 1,112 samples (Figure 2B) and used the 872 original samples to compute the average value of PC1 and PC2 for each region *r*. For each of the 250 additional samples, we computed the Euclidean distance between (PC1,PC2) for that sample and the average value of (PC1,PC2) for region *r*, and defined our prediction of regional ancestry as the value of *r* minimizing that distance. We defined true ancestry as the region in which the greatest number of ancestors five generations back was born. We compared predicted ancestry with true ancestry, both for the set of 250 samples and for a subset of 98 samples with majority ancestry from a single region. Given the low number of ancestors from region 1 (see Table 1), we merged region 1 with region 11 in these analyses (see Figure 1). This had little effect on our results, as only two of the 250 samples and none of the subset of 98 samples had the greatest number of ancestors from region 1. Thus, predicted ancestry *P* and true ancestry *T* each had values between 2 and 11. We considered our ancestry prediction to be correct if 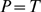, correct to within a distance of one region if 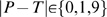, and correct to within a distance of two regions if 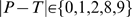 (see Figure 1).

### Samples from Norway and Scotland

The Icelandic population arose from the admixture of Norse and Gaelic ancestors. To represent the ancestral populations, 445 samples from Scotland were genotyped on the Illumina 300 K chip, and 250 samples from Norway were genotyped on the Affymetrix 6.0 chip. The appropriate informed consent was obtained for all sample donors. Illumina 300 K genotyping was conducted by deCODE Genetics, and Affymetrix 6.0 genotyping was conducted by Expression Analysis on behalf of Ulleval University Hospital in Oslo. SNPs with >5% missing data in either Norway or Scotland were removed, leaving 79,641 autosomal SNPs (that were genotyped on both chips) in the merged data set of samples from Iceland, Norway and Scotland.

### Assessment of Nordic and Gaelic Ancestry in the Icelandic Population

Let *N_j_* and *p_j_* denote total allele count and observed allele frequency in the Icelandic population, *N_j_*_1_ and *p_j_*_1_ denote total allele count and observed allele frequency in ancestral population 1, and similarly *N_j_*_2_ and *p_j_*_2_ in ancestral population 2, for SNP *j*. Let MIX*_α_* denote a synthetic population consisting of a linear combination of proportions *α* and (1−*α*) from ancestral populations 1 and 2, respectively. Let *p_jα_* = *α p_j_*_1_+(1−*α*) *p_j_*_2_. We estimate the *F*_ST_ between Iceland and MIX*_α_* as
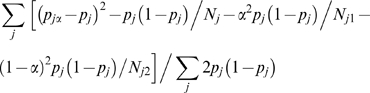
with the subtracted terms in the numerator adjusting for the effects of sampling error (see Supp Note 10 of [34]). We note that linkage disequilibrium between SNPs may lead to suboptimal weighting, which will increase the variance but will not bias the estimate. We estimate *F*_ST_ for different values of *α* (on an evenly spaced grid from 0 to 1) and infer the ancestry proportion *α* that minimizes *F*_ST_, as described previously [35],[36]. We compute the standard error of the ancestry estimate *α* via a bootstrap approach. We partition the set of SNPs into *B* disjoint blocks (e.g., *B* = 100), repeat the computation for SNPs in each block to obtain *B* different ancestry estimates, and compute the standard error as the standard deviation of these estimates divided by the square root of *B*. Standard errors of *F*_ST_ estimates are computed in the same way. We note that the computation of *F*_ST_ between two sampled populations is equivalent to the above formula for *α* = 0 or *α* = 1.

Our *F*_ST_ computations assume that allele frequencies are obtained from an unrelated set of individuals. If related individuals were used, the effects of sampling error would be underestimated. Unrelated individuals were used in all *F*_ST_ computations, except in analyses of the aggregate set of Icelandic individuals, which included some related pairs of individuals. In this analysis, we used a subset of 30,244 of the 35,457 Icelandic individuals genotyped, in which the most closely related samples were removed. In this case, the amount by which the estimated sampling error (equal to the reciprocal of *N* = 2×30,244) is inaccurate is expected to be far smaller than the precision of 0.0001 to which we report *F*_ST_ estimates, and hence negligible.

### Distribution of Allele Frequency Differences

Under neutral drift, the difference (*p*_1_−*p*_2_) between observed allele frequencies of two populations at a given locus can be approximated as a normal distribution with mean 0 and variance *p*(1−*p*)(2*F*_ST_+1/*N*_1_+1/*N*_2_), where *F*_ST_ is the genetic distance between the two populations, *N*_1_ and *N*_2_ are total allele counts in each population, and *p* is the ancestral allele frequency that can be approximated as the average of the two observed allele frequencies [37]. We note that this null model extends to the case of admixture, which simply scales *F*_ST_ by the square of the admixture coefficient. It follows that (*p*_1_−*p*_2_)^2^/[*p*(1−*p*)(2*F*_ST_+1/*N*_1_+1/*N*_2_)] is χ^2^ distributed with 1 degree of freedom (d.o.f.). In fact, one can simply compute (*p*_1_−*p*_2_)^2^/[*p*(1−*p*)] divided by its mean across SNPs, avoiding complications involving the effective sample size in the case of related samples. In these computations we excluded SNPs with minor allele frequencies *p*<0.05 to minimize deviations from the normality assumption. An excess of large values of the χ^2^ statistic indicates deviations from the null model, suggesting the action of natural selection.

Relationship between the distributions of allele frequency differences and disease association statistics, if cases and controls are drawn from distinct populations. We provide a mathematical derivation for the result that a null distribution of allele frequency differences implies a null distribution of disease association statistics after correction by genomic control. We consider a hypothetical association study in which *N*/2 diploid disease cases are drawn from population 1 and *N*/2 diploid controls are drawn from population 2. Any instance of population stratification can be considered in this framework by defining population 1 and population 2 as appropriate admixtures of the underlying populations. For a given marker, let *p*_1_ and *p*_2_ denote observed frequencies in cases and controls and *p* be the mean of *p*_1_ and *p*_2_. It follows that the correlation between genotype and case-control status is equal to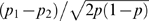, so that the Cochran-Armitage trend statistic [38], which equals *N* times the square of that correlation, is equal to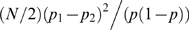. Since (*p*_1_−*p*_2_) is normally distributed with mean 0 and variance *p*(1−*p*)(2*F*_ST_+1/*N*_1_+1/*N*_2_), where *N*_1_ = *N*_2_ = *N* (see above), it follows that the Cochran-Armitage trend statistic has a χ^2^ (1 d.o.f.) distribution scaled by (1+*NF*_ST_). (See [39] for a related derivation.) This means that when the method of genomic control [40] is applied, the inflation factor *λ* is equal to 1+*NF*_ST_, and that dividing association statistics by *λ* results in a χ^2^ (1 d.o.f.) distribution. More generally, the fact that both the allele frequency difference statistic and the Cochran-Armitage trend statistic are proportional to (*p*_1_−*p*_2_)^2^/(*p*(1−*p*)) implies that the distributions of these two statistics are identical up to a constant scaling factor, even when allele frequency differences do not follow a null distribution.

## Supporting Information

Figure S1 PCA plot of 203 samples with ancestry from 11 regions of Iceland projected onto PCs computed using 674 nonoverlapping Icelandic samples.
(0.14 MB TIF)

Figure S2 Joint PCA plots of 877 Icelandic, 250 Norwegian and 445 Scottish samples. We plot (a) the top two PCs and (b) the third and fourth PCs.
(0.42 MB TIF)

Table S1 Iceland, Scotland, Norway and HapMap allele frequencies of markers from Table 3.
(0.04 MB DOC)

Text S1 Supplementary note.
(0.03 MB DOC)
