# Supplementary figures and images for: The Impact of Divergence Time on the Nature of Population Structure: An Example from Iceland
Source: PLoS Genet. 2009 Jun 5;5(6):e1000505. doi: 10.1371/journal.pgen.1000505 (PMC2684636; doi:10.1371/journal.pgen.1000505)

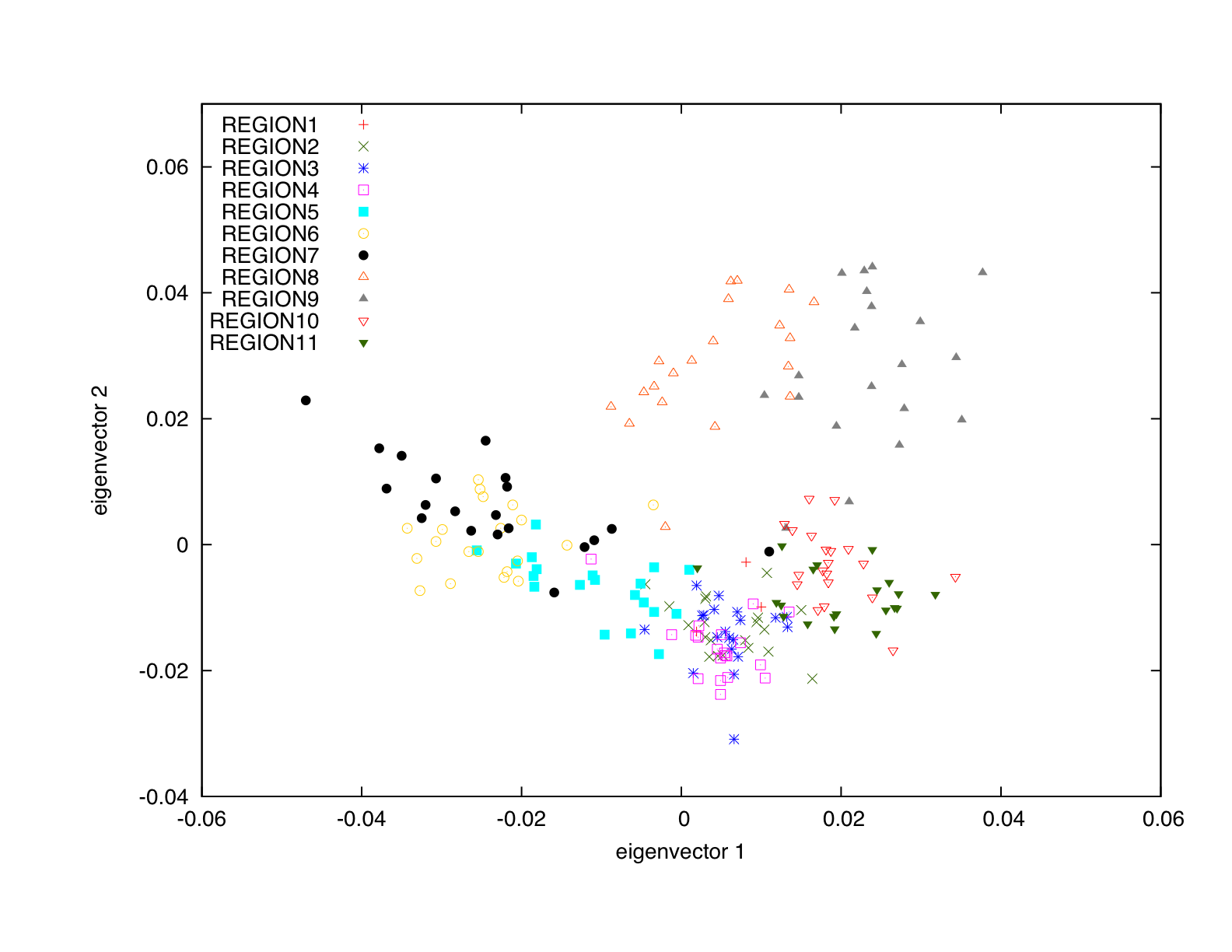

Supplement: Figure S1 — PCA plot of 203 samples with ancestry from 11 regions of Iceland projected onto PCs computed using 674 nonoverlapping Icelandic samples. (0.14 MB TIF) [file pgen.1000505.s001.tif]

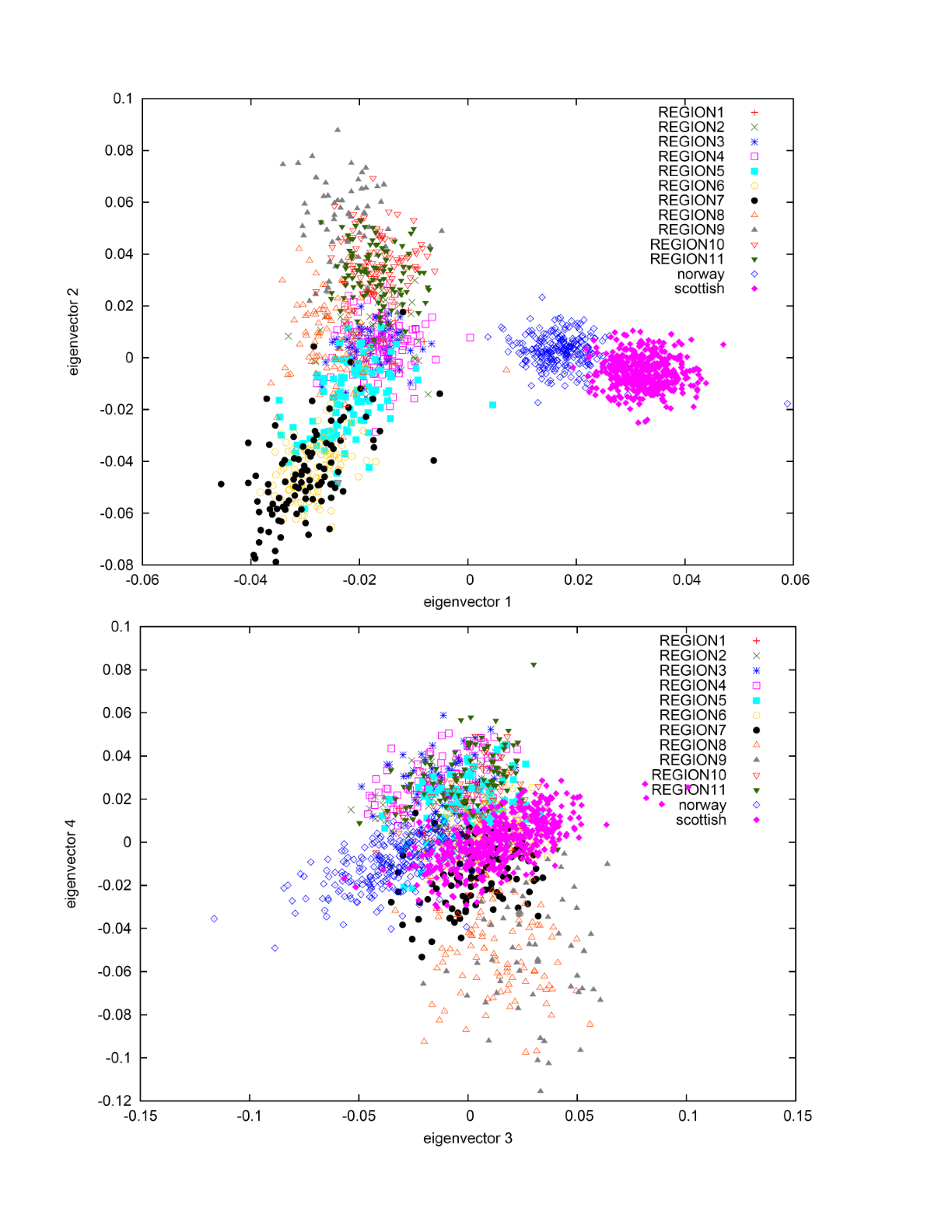

Supplement: Figure S2 — Joint PCA plots of 877 Icelandic, 250 Norwegian and 445 Scottish samples. We plot (a) the top two PCs and (b) the third and fourth PCs. (0.42 MB TIF) [file pgen.1000505.s002.tif]
