# Supplementary material for: The Impact of Divergence Time on the Nature of Population Structure: An Example from Iceland
Source: PLoS Genet. 2009 Jun 5;5(6):e1000505. doi: 10.1371/journal.pgen.1000505 (PMC2684636; doi:10.1371/journal.pgen.1000505)
Supplement: Table S1 — Iceland, Scotland, Norway and HapMap allele frequencies of markers from Table 3. (0.04 MB DOC) [file pgen.1000505.s003.doc]

Supplementary Table

Table S1. Iceland, Scotland, Norway and HapMap allele frequencies of markers from Table 3. We list frequencies in each population of the allele that is the minor allele in Europeans. Markers not genotyped on the Affymetric 6.0 chip have Norway frequencies listed as n/a.

| marker | Iceland | Scotland | Norway | CEU | YRI | CHB | JPT |
| --- | --- | --- | --- | --- | --- | --- | --- |
| rs10024216 | 0.38 | 0.21 | 0.38 | 0.42 | 0.33 | 0.68 | 0.69 |
| rs10008492 | 0.43 | 0.22 | n/a | 0.43 | 1.00 | 1.00 | 1.00 |
| rs4331786 | 0.40 | 0.20 | n/a | 0.41 | 0.44 | 0.72 | 0.73 |
| rs11096957 | 0.40 | 0.20 | 0.39 | 0.41 | 0.51 | 0.70 | 0.69 |
| rs4543123 | 0.30 | 0.11 | n/a | 0.30 | 0.58 | 0.58 | 0.44 |
| rs4833095 | 0.30 | 0.12 | n/a | 0.30 | 0.87 | 0.69 | 0.64 |
| rs7944926 | 0.35 | 0.17 | n/a | 0.27 | 0.80 | 0.51 | 0.73 |
| rs3794060 | 0.36 | 0.17 | n/a | 0.27 | 0.84 | 0.52 | 0.73 |
| rs13107325* | 0.02 | 0.11 | n/a | 0.12 | 0.00 | 0.00 | 0.00 |
