# Supplementary material for: The Impact of Divergence Time on the Nature of Population Structure: An Example from Iceland
Source: PLoS Genet. 2009 Jun 5;5(6):e1000505. doi: 10.1371/journal.pgen.1000505 (PMC2684636; doi:10.1371/journal.pgen.1000505)
Supplement: Text S1 — Supplementary note. (0.03 MB DOC) [file pgen.1000505.s004.doc]

Supplementary Note

Genetic relationships between 11 regions of Iceland

We investigated whether the genetic patterns we observed (Figure 2a) were the result of genome-wide structure, or of a small number of highly informative markers. We used EIGENSOFT 2.0 to compute SNP loadings for each of the top two PCs, normalized to mean 0 and standard deviation 1. Under a normal distribution, we would expect 0.01% of markers (29 markers) to have a SNP loading with absolute value greater than 3.89, which corresponds to a P-value of 0.0001. We actually observed that 0.009% markers (26 markers) exceeded this threshold for PC1 and 0.013% of markers (38 markers) exceeded this threshold for PC2, similar to what would be expected under a normal distribution. For both PC1 and PC2, less than 1% of the variance of that PC was explained by the top 100 markers for that PC (estimated proportions were 0.49% for PC1 and 0.52% for PC2; these numbers are overestimates because we did not account for linked markers in this computation). Thus, the top two PCs are clearly the result of genome-wide structure, rather than being driven by a small number of highly informative SNPs.

Genetic relationships between Iceland, Norway and Scotland

A potential concern with the analysis of Figure 3, in which Norwegian and Scottish samples were projected onto PCs computed using 877 Icelandic samples, is that projected samples may be affected by regression towards the mean if samples used to compute PCs have insufficient sample size. To evaluate this concern, we excluded 203 of the 877 Icelandic samples (all 3 samples from region 1 plus 20 samples from each other region), computed PCs using the remaining 674 samples, and projected the 203 samples onto those PCs. Results are displayed in Figure S1, which indicates that the 203 projected samples exhibit the same patterns of variation as in Figure 2a. We do however note that the scale of Figure S1 is somewhat smaller than the scale of Figure 2a. This is a function of the sample size of the samples used to compute PCs: with large sample size, SNP loadings are extremely accurate and no regression towards the mean will occur in projected samples; with smaller sample size, SNP loadings become less accurate (even if the PCs themselves are accurate) and regression towards the mean will occur.

We also considered a joint PCA of 877 Icelandic, 250 Norwegian and 445 Scottish samples in which all samples were used to compute PCs (Figure S2a). To first approximation, the top PC represents between-country variation and the second PC represents within-Iceland variation. Lower PCs show a similar pattern (Figure S2b). These results are concordant with Figure 3. However, we consider the joint PCA plots of Figure S2 to be less informative, because a single principal component may aggregate multiple effects in order to maximize variation captured. For example, the slightly more extreme position of region 9 along the top PC of Figure S2a need not be related to the fact that this axis separates Norway and Scotland, but may instead reflect a larger amount of Iceland-specific drift in region 9 (see main text).

Distribution of allele frequency differences between Iceland and Scotland

As noted in the main text, we used Icelanders and Scots (rather than Norwegians) in this analysis, because tail distributions of comparisons of populations genotyped on different chips appear to be confounded by assay artifacts. For example, for SNP rs888781 we observed frequencies 32% in Iceland (Illumina 300K), 30% in Scotland (Illumina 300K) and 94% in Norway (Affymetrix 6.0). In HapMap populations, this SNP has frequencies 27% in CEU, 14% in YRI, 25% in CHB and 28% in JPT. We hypothesize that the vastly different frequency in Norway is an artifact of Affymetrix 6.0 genotyping rather than a true population difference.
